# Supplementary material for: Best Evidence to Best Practice: Implementing an Innovative Model of Nutrition Care for Patients with Head and Neck Cancer Improves Outcomes
Source: Nutrients. 2020 May 19;12(5):1465. doi: 10.3390/nu12051465 (PMC7284331; doi:10.3390/nu12051465)
Supplement: Supplementary file 1 [file nutrients-12-01465-s001.pdf]

Supplementary Table S1. Team-level evaluation—multidisciplinary team focus group semi-structured question schedule.

Question Schedule
**1.** What are your overall impressions of the new nutrition care strategies that have been put in place as part of the new model of care?
**2.** How did you find the Supportive Care-Led Pre-Treatment Clinic?<br>**a.** Were there any benefits? Drawbacks?<br>**b.** Were there any barriers? Facilitators?<br>**c.** Do you feel this was an appropriate strategy for this group of patients?<br>**d.** Is this something you would be happy to adopt as standard care?<br>**e.** Is the pre-treatment clinic offered at the right time?<br>**f.** Can you tell me a little bit about your experience with using the nutrition assessment tool (PG-SGA [a])?<br>**g.** Did that change over time?
**3.** How did you find the Nutrition Care Dashboard?<br>**a.** Were there any benefits? Drawbacks?<br>**b.** Were there any barriers? Facilitators?<br>**c.** Do you feel this was an appropriate strategy for this group of patients?<br>**d.** Is this something you would be happy to adopt as standard care?<br>**e.** How useful is it having nutrition outcome measures (weight change, PG-SGA) available in real-time?<br>**f.** Can you tell me a little bit about your experience with having the new dietitian documents available in the eMR [b]?
**4.** Have you changed your practice as a result of the new nutrition care strategies?<br>**a.** In what ways?<br>**b.** Was there anything particularly helpful/not helpful?<br>**c.** Has there been any impact on your professional practice, e.g., confidence?
**5.** What is your sense of how these strategies have impacted on patients'/caregivers' experience?<br>**a.** Can you give any examples?<br>**b.** How acceptable do you think the new model of care is to patients/caregivers?
**6.** What are your impressions of how sustainable these strategies are in the longer term?<br>**a.** Are there any risks of it not continuing?<br>**b.** What do you think are the main benefits of it continuing?<br>**c.** Do you feel there is a sense of ownership for these strategies within the MDT?<br>**d.** Is there clinical leadership in place for sustaining these strategies into the future?
**7.** Do you have any other comments or thoughts you'd like to share?<br>**a.** Do you have any suggestions as to how to improve the model of care?<br>**b.** Is there anything you think worked well/didn't work well?

[a] PG-SGA = Patient-Generated Subjective Global Assessment; [b] eMR = electronic medical record.
